# Knowledge and practices of health practitioners on treatment of Buruli ulcer in the Mbonge, Ekondo Titi and Muyuka Health Districts, South West Region, Cameroon

**DOI:** 10.11604/pamj.2018.31.228.17420

**Published:** 2018-12-10

**Authors:** Frankline Sanyuy Nsai, Samuel Nambile Cumber, Ngwayu Claude Nkfusai, Vecheusi Zennobia Viyoff, Nkemngu Blake Afutendem, Rosaline Yumumkah Cumber, Joyce Mahlako Tsoka-Gwegweni, Jane-Francis Tata Kihla Akoachere

**Affiliations:** 1 Department of Public Health and Hygiene, University of Buea, Buea, Cameroon; 2 Section for Epidemiology and Social Medicine, Department of Public Health, Institute of Medicine, the Sahlgrenska Academy at University of Gothenburg, Gothenburg, Sweden; 3 Faculty of Health Sciences, University of the Free State, Bloemfontein, South Africa; 4 Department of Microbiology and Parasitology, Faculty of Science, University of Buea, Buea, Cameroon; 5 Department of Public Health, Faculty of Science, University of Dschang, Dschang, Cameroon; 6 Department of Political Science, University of KwaZulu-Natal, Durban, South Africa; 7 School of Nursing & Public Health, College of Health Sciences, University of KwaZulu-Natal, Durban, South Africa

**Keywords:** Buruli Ulcer, knowledge, practices, treatment, health practitioners, health districts

## Abstract

**Introduction:**

After tuberculosis and leprosy, Buruli ulcer (BU) is the third most common mycobacterial infection. Buruli ulcer begins as a localized skin lesion that progresses to extensive ulceration thus leading to functional disability, loss of economic productivity and social stigma. This study is aimed at assessing the knowledge and practices among health practitioners on the treatment of BU in the Mbonge, Ekondo Titi and Muyuka Health Districts of the South West Region of Cameroon.

**Methods:**

This is a cross-sectional study that investigates participants' knowledge and practices on the treatment of BU. The study uses a qualitative method of structured questionnaires in the process of data collection.

**Results:**

Seventy percent (70%) of the participants acknowledged they encounter cases of BU in their respective Hospitals or Health centers. Among these, 48% agreed they managed BU in their facilities and up to 91.7% noted that their community members are aware that BU is managed in their facility while seventy percent of the medical practitioners indicated they cannot identify the various stages of BU. Eighty-one percent of the practitioners from Muyuka HD indicated they could not identify the various stages of BU. More than 63% of the practitioners regarded BU patients as normal people in their communities however, practitioners that practiced for less than 5 years were likely not to admit BU patients in the same room with other patients. Beliefs such as being cursed (47.06%) and being possessed (29.41%) were reported by practitioners that acknowledged the existence of traditional beliefs in the community.

**Conclusion:**

Despite the fact that a majority of the health practitioners knew what BU is, most of them demonstrated lack of knowledge on the identification of the various stages and management of the illness. Practitioners demonstrated positive attitude towards patients although they would not admit them in the same room with other patients. Considering the poor knowledge on identification and management demonstrated by most of the practitioners, management of the disease would be inadequate and may even aggravate the patient's situation. Training and onsite mentorship on screening, identification and management of BU is therefore highly recommended amongst health personnel practicing in endemic areas.

## Introduction

After tuberculosis and leprosy, Buruli ulcer (BU) is the third most common mycobacterial infection [1]. BU begins as a localized skin lesion that progresses to extensive ulceration, leading to functional disability, loss of economic productivity, and social stigma [2]. In Australia BU is called Brainsdale disease. Since the 1998 World Health Organization (WHO) Buruli ulcer initiative, there has been increased attention to research efforts for treatment and control of BU. Buruli ulcer, Bairnsdale ulcer and Daintree ulcer are all local names given to the same disease that is caused by *Mycobacterium ulcerans*. The responsible organism is an acid-fast *Mycobacterium* of the same genus as the tuberculosis bacilli. This environmental bacterium produces a destructive toxin and mycolactone which leads to tissue damage that inhibits the immune response [3]. *M. ulcerans* infects the skin and subcutaneous tissues that progresses to indolent non-ulcerated and ulcerated lesions [4]. *M. ulcerans* grows optimally at a temperature of 90oF (32°C) in the tropical and sub-tropical zones on earth [5]. Considering Cameroon as one of the most affected areas in Africa, there has been intense study on various aspects of this debilitating disease in the country. Prevalence studies of BU by [6] and [7] in the Nyong River basin in Akonolinga identified 436 and 125 cases of BU respectively. Also, studies by [8] show that the age adjusted cumulative incidence in elderly people is similar to that in children because of contact with the river which is a risk factor. The current Bacille Calmette-Guérin (BCG) vaccine appears to offer some short-term protection against BU however, there is on-going research for a vaccine to treat Buruli ulcer because a safe and effective vaccine may be the most effective way to combat Buruli ulcer in the long term. Although the first report of Buruli ulcer from Africa dates back to 1897 when Sir Albert Cook described cases of chronic ulceration in Uganda, the first definitive description of *Mycobacterium ulcerans* was published in 1948 [4].

Cases of human disease occur in over 30 countries worldwide. For instance, disease foci have been reported from tropical areas in Asia (Malaysia, Papua New Guinea and Sri Lanka) and Latin America (Guyana, Mexico, Peru). In addition, the largest numbers of patients with Buruli ulcer disease have been detected in sub-Saharan Africa [9]. Foci have also been identified in Australia (Queensland and the Southern Territory) [10]. The earliest reports from Africa came from the South-West of Kinshasa in the Democratic Republic of the Congo where the disease is prevalent [11]. Later, there were reports of hundreds of patients in Uganda, from a refugee camp in Kinyara, near the Nile river, in a county that was called Buruli, hence the name “Buruli ulcer” [12]. The last training on BU offered to health care providers in the SWR was in 2007 thus indicating that a high proportion of health care providers (medical doctors, nurses and laboratory technicians), especially those recruited after the training could have little or no knowledge on the identification and management of BU. It is therefore evident that there is need for evaluation of knowledge and practices of health care providers in the SWR of Cameroon to make recommendations on ways to facilitate disease identification and prompt treatment to minimize the physical, economic and social impacts of the disease. The major objective of this study was to assess the knowledge of medical practitioners about BU with regards to its transmission, prevention and treatment, and also to determine their behavior towards BU disease sufferers.

## Methods

**Study design:** this was a cross sectional study that involved principally the administration of questionnaires to a cross-section of the participants (nurses, doctors and laboratory technicians) in the Mbonge, Ekondo Titi and Muyuka Health Districts (HDs) to investigate participants' knowledge and practices on the treatment of BU. Three Health Districts with high prevalence of BU were selected from 18 Health Districts in the South West Region.

**Study area:** the South West region is made up of 18 HDs and this study covered three HDs (Ekondo Titi, Mbonge and Muyuka) based on the prevalence of Buruli ulcer in these Health Districts.

### Sample size calculation

$$\text{n}=\left[\begin{array}{c} \underline{\text{P}(1-\text{p})}\\ \frac{{\text{A}}^{2}+}{{\text{ Z}}^{\text{2}}}\;\frac{\text{P}(1-\text{P})+}{\text{ N}}\\ \bar{\;R\;} \end{array}\right]$$

Where: n = Sample size required N = Number of health workers practicing in the concerned health facilities in the population: (100) P = Estimated variance in population, as a decimal: (0.5 for 50-50) A = Precision desired, expressed as a decimal: (0.05 for 5%) Z = Confidence level: 1.96 for 95% confidence R = Estimated Response rate, as a decimal: (1) Applying the formula:

$$\text{n}=\frac{\begin{array}{c} \underline{0.5(1-0.5)}\\ \frac{0.05^{2}}{1.96^{2}}\text{ +}\frac{0.5(1-0.05)}{100} \end{array}}{1}$$

n = 79.34 respondents and it was increased to 80 respondents. Twenty respondents from each health district (80 respondents divided by 4 HDs). Four health workers were to be evaluated from each health facility.

### Selection criteria

**Inclusion criteria:** healthcare providers' resident in the health districts who accepted to participate and gave their consent; healthcare providers involved in the Neglected tropical diseases (NTDs) program; all sexes, 21yrs to 60yrs.

**Exclusion criteria:** health personnel not resident in the Health District; health personnel not involved in the NTDs Program; age less than 21 years.

**Study population:** the study population was made up of health practitioners from three Health Districts implementing the NTDs program.

**Research design and data collection:** this study was a cross-sectional survey, in which participants' knowledge and practice on BU was investigated. Data was obtained quantitatively through structured questionnaires.

**Questionnaires:** questionnaires that had initially been tested and validated were administered to medical practitioners. This questionnaire had three sections: demographics, understanding and treatment seeking behavior of the disease and finally practice on the BUD (Buruli Ulcer Disease) sufferers. These questionnaires aimed at determining what participants (nurses, doctors and laboratory technicians) know about BU with regards to its transmission, prevention and treatment. In addition, the questionnaires were aimed at establishing the behavior of the practitioners towards sufferers.

**Data analysis:** the statistical package EPI Info 7 was used to enter data in this study. SPSS version 20.0, statistical software was used in data cleaning, management and analysis. A descriptive analysis on the cases was done. The relationship between the study outcome (knowledge and practice of practitioners and the independent (professional qualification, location of health facility and work experience) variables was analyzed using the chi-square test.

**Ethical considerations:** ethical clearance for this study was obtained from the Institutional Review Board of the Faculty of Health Sciences, University of Buea. Also, administrative clearance was obtained from the RDPH of the South West region. At the study sites, consent was sought from the district medical officers while study participants signed an informed consent form to willingly participate in the study.

## Results

**Figure 1:**
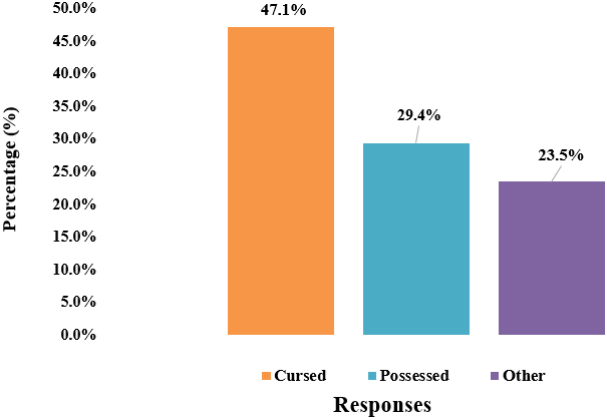
Identification and management of BU by practitioners

**Figure 2:**
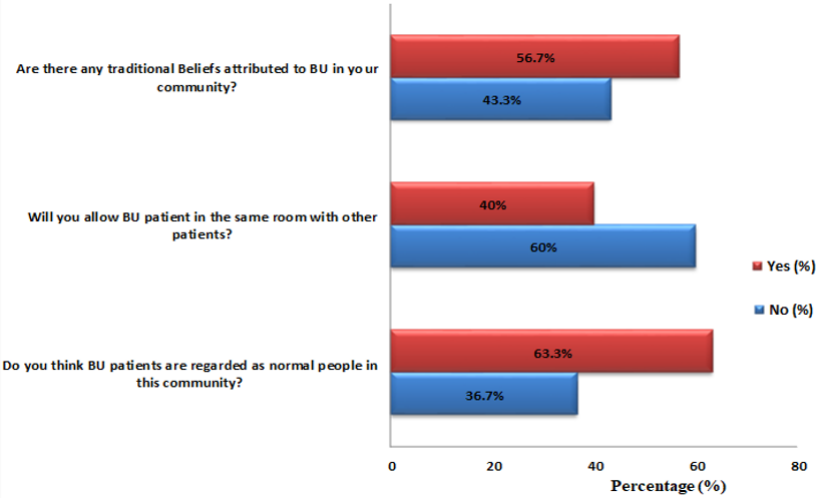
Frequency presentation of BU cases in health facilities

**Figure 3:**
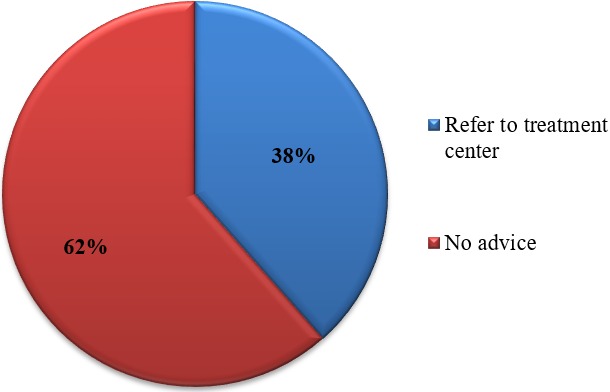
Advice given to patients by practitioners

**Figure 4:**
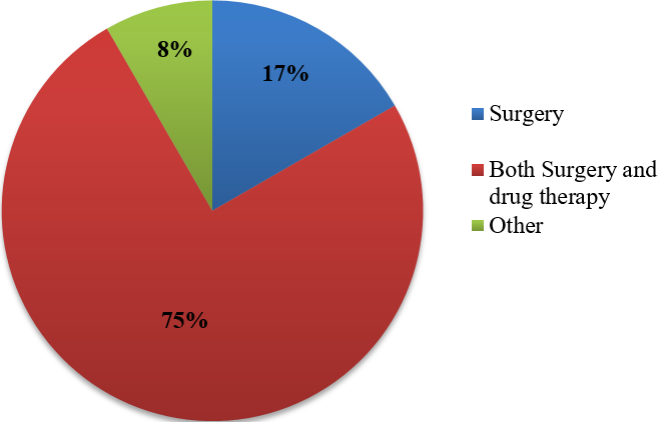
Best method used to treat BU

**Knowledge and practices of health practitioners on treatment of BU:** seventy percent (70%) of the participants acknowledged they encounter cases of BU in their respective Hospitals/Health centers. Among these, 48% agreed they managed BU in their facilities and up to 91.7% noted that their community members are aware that BU is managed in their facility. Seventy percent of the medical practitioners indicated they cannot identify the various stages of BU (Figure 1). Stratification of the participants with respect to various Hospitals/Health Centers gave a better understanding about knowledge on treatment of BU. Muyuka District Hospital was likely to encounter BU cases in their facility and were less likely to manage BU cases. Averagely, 81.25% of the practitioners from Muyuka HD indicated they could not identify the various stages of BU. All (100%) health practitioners from District hospital Ekondo Titi, Kumbe Balue Health Center Ekondo Titi, Bokosso Health Center, District Hospital Muyuka and Calvary Hospital Muyuka admitted receiving cases of BU in their facility. Also, all practitioners in the District Hospital of Ekondo Titi and Kumbe Balue Health Center Ekondo Titi indicated that their community members are aware they managed the disease. With respect to identification of various stages of the disease, there was no facility where all practitioners could identify the stages of BU. Eighty percent (80%) of the practitioners in DH Ekondo Titi could identify the various stages of BU (Table 1). Of the practitioners that acknowledged they encounter BU cases in their respective facilities, 60% of them encountered BU often while 40% encountered it occasionally (Figure 2). Thirty-eight percent of the participants that indicated they don't manage BU cases in their health facilities referred the patients to treatment centers while 61.5% gave no advice to the patients. For participants that agreed they manage BU, 75% indicated both drug therapy and surgery as best methods for treatment while 16.7% thought that having a surgery could be the only way to treat BU (Figure 3, Figure 4).

**Table 1:** Knowledge on treatment of BU in various health districts

Name of Facility	N (%)	Do you Encounter Cases of BU in Your facility? (%)		Do you manage BU cases in your facility? (%)		Are your Community Members aware that you manage BU cases in your Facility? (%)		Can you identify the various stages of BU? (%)	
		No	Yes	No	Yes	No	Yes	No	Yes
	80(100)	39.2	64.4	77.3	22.7	18.8	81.3	78.3	21.7
District Hospital Ekondo Titi	13(16.67)	0	100	0	100	0	100	20	80
Pamol Hospital Lobe	8(10)	66.7	33.3	100	0	N/A	N/A	66.7	33.3
Kumbe Balue Health Center Ekondo Titi	5(6.67)	0	100	50	50	0	100	100	0
Bokosso Health Center	5(6.67)	0	100	100	0	N/A	N/A	100	0
District Hospital Mbonge	12(13.33)	100	0	0	100	25	75	50	50
Kotto Barombi Health Center	5(6.67)	50	50	100	0	N/A	N/A	100	0
Kombone Health Center	5(6.67)	50	50	100	0	N/A	N/A	100	0
Little Angles Muyuka	5(6.67)	25	75	100	0	N/A	N/A	100	0
District Hospital Muyuka	12(13.33)	0	100	100	0	N/A	N/A	75	25
Healing Touch Medical Center	5(6.67)	100	0	100	0	50	50	50	50
Calvary Health Services Muyuka	5(6.67)	0	100	100	0	N/A	N/A	100	0

N/A means not applicable

**Figure 5:**
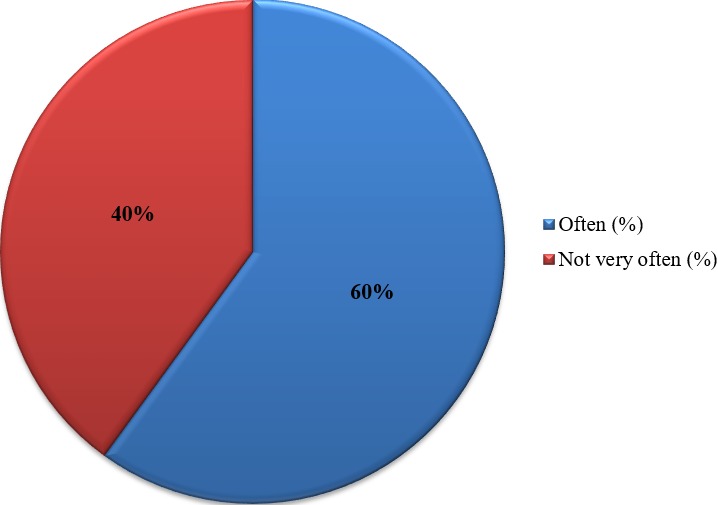
Attitude and perception of practitioners towards BU patients

**Figure 6:**
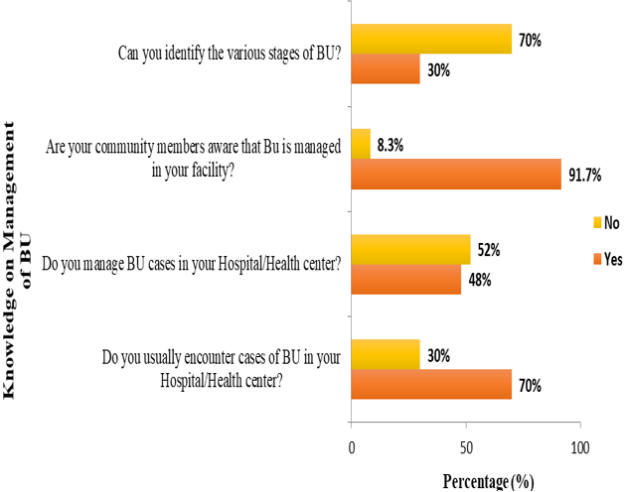
Practitioners opinion on beliefs attributed to BU existing in the community

**Attitude and perception of health practitioners towards BU patients:** over 63% of the practitioners regarded BU patients as normal people in their communities however, 60% percent of them will not admit BU patients in the same room with other patients. Additionally, more than 56% of the practitioners reported the existence of traditional beliefs attributed to BU in their respective communities (Figure 5). Stratification with respect to demographic factors of the practitioners revealed some aspects about attitudes and perception towards patients. For instance, health practitioners between the ages of 25 and 40 would likely not admit BU patients in the same room with other patients. At the same time, practitioners that had not practiced for more than 5years were also likely not to admit BU patients in the same room with other patients. Focusing on profession, there was no significant difference in knowledge and attitude towards patients (Table 2). The following beliefs were reported by practitioners that acknowledged the existence of traditional beliefs in the community: curse (47.06%) and being possessed (29.41%). Twenty-four percent of the practitioners thought there were other beliefs attributed to BU in their respective communities. For instance, beliefs like witchcraft and totems (snakes) were noted (Figure 6).

**Table 2:** Knowledge and attitude of health practitioners towards BU patients

Demographics	N (%)	Are BU patients regarded as normal people in this community? (%)		Would you admit BU patients in the same room with other patients? (%)		Are there any traditional beliefs attributed to BU in this community? (%)	
	80(100)	No	Yes	No	Yes	No	Yes
**Sex**							
Male	40(50)	33.3	66.7	60	40	40	60
Female	40(50)	40	60	60	40	46.7	53.3
**Age**							
25-40	50(63.3)	31.6	68.4	73.7	26.7	42.1	57.9
41-55	24(30.0)	55.6	44.4	55.6	44.4	33.3	66.7
56+	6(6.7)	0	100	0	100	0	100
**Profession**							
Nurse	50(63.3)	36.8	63.2	63.2	36.8	42.1	57.9
Medical Doctor	14(16.7)	40	60	20	80	43.3	56.7
Laboratory Technician	16(20.0)	33.3	66.7	83.3	16.7	33.3	66.7
**Length of time in service**							
Less than 1yr	8(10.0)	66.7	33.3	100	0	33.3	67.7
Between 1yr and 5yrs	7(23.3)	28.6	71.4	100	0	42.9	57.1
More than 5yrs	20(66.7)	35	65	40	60	45	55
**Time spent in Hospital/Health center**							
Less than 1yr	14(16.7)	40	60	80	20	40	60
Between 1yr and 5yrs	37(46.7)	35.7	64.3	64.3	35.7	28.6	71.4
More than 5yrs	29(36.7)	27.3	72.7	45.5	54.5	63.6	36.4

## Discussion

Among the health practitioners, out of the 70% that acknowledged they usually encounter BU cases in their health facilities, surprisingly, less than one-third of the practitioners confirmed that they could identify the various stages of BU. 60% of the practitioners also thought BU can be prevented by citing specific possible means through which the debilitating illness can be prevented. According to information obtained from community members during FGDs in a related study and in consideration of the fact that a handful of the health practitioners were unable to identify and manage BU cases in their health facilities, they were tempted to seek treatment elsewhere. They mentioned prayers (miracles) and herbalists/witchdoctors as alternative places for treatment [13]. With the last effective training on the identification and management of BU in the SWR dating back as far as 2007 [14], newly recruited practitioners or those transferred from non-endemic to endemic areas, (especially nurses) lacked knowledge on the disease hence, management errors are mentioned above since they have not had any formal training on identification and management. These are indications that there is lack of knowledge of disease management. It is worth noting that in Muyuka where no treatment center exists, 38% of practitioners reported they would refer suspected cases to treatment centers.

The SWR has only two treatment centers for BU which are based in Ekondo titi HD and Mbonge HD respectively. These centers are located far from other HDs such as the Muyuka HDs that often report more on BU cases. A study by Akoachere *et al* in 2016 [13] on community members revealed that; since treatment centers are relatively far from other HDs, community members preferably sought treatment elsewhere such as going to herbalists/witchdoctors. The study further indicated that apart from the distance which is a limiting factor, the poor state of roads in these areas equally prevented patients from traveling to treatment centers. Among the health practitioners, 60% of them would not admit BU patients in the same room with other patients. Also, practitioners that had been in service for less than 5 years were more likely to think that BU patients should not be admitted in the same room with other patients. This attitude additionally indicates that there are gaps in the knowledge and management of the disease by practitioners. Considering the length of time (10 years) since the last regional training on BU was carried out in the SWR, many practitioners have either joined the profession or have been transferred to this region and it is obvious from the results that hands full of them are ignorant about the disease. Medical practitioners also confirmed that there were traditional beliefs like witchcraft, being cursed and being possessed that are attributed to the disease by the community. These findings demonstrate that there is lack of information about the disease in endemic localities that generally affects management.

## Conclusion

From the findings presented above, although participants demonstrated awareness on BUD, there is still a poor knowledge on the disease. Among those that had knowledge about the disease, majority of them knew it is due to bacterial infection. The hospital was acknowledged as the ideal place for treatment even though some of participants preferred other sources like herbalist/witchdoctors or prayers. Longevity in service further played an important role in the knowledge on BU and attitude towards patients because despite the fact that most of the health practitioners usually encounter BU cases, few of them normally referred the patients to treatment centers. Considering the poor knowledge on identification and management demonstrated by most of the practitioners, management of the disease becomes inadequate thus aggravating the patient's situation. Consequently, reinforcement of BU identification and management in the curriculum for training medical practitioners is important as this may go a long way to increase their knowledge on BU as well as help in taming their attitude and perceptions towards patients.

### What is known about this topic

- Knowledge by members of affected communities and attitude and practice towards patients;
- Epidemiology and socio-economic effects of BU among members of affected communities.

### What this study adds

- Shows that health practitioners in endemic localities lack knowledge on the management of BUD, despite its endemicity;
- Demonstrates a need for training of practicing professionals on BU identification and management and also the inclusion of this topic in the curriculum of health students.

## Competing interests

The authors declare no competing interests.
